# How to Treat a Child With a Concurrent Diagnosis of Leukemia and Generalized Mucormycosis? Case Report

**DOI:** 10.3389/fmed.2022.844880

**Published:** 2022-02-25

**Authors:** Patrycja Sosnowska-Sienkiewicz, Kinga Błaszczyk, Honorata Kubisiak-Rzepczyk, Przemysław Mańkowski, Danuta Januszkiewicz-Lewandowska

**Affiliations:** ^1^Department of Pediatric Surgery, Traumatology and Urology, Poznan University of Medical Sciences, Poznan, Poland; ^2^Department of Medical Diagnostic, Poznan, Poland; ^3^Department of Dermatology, Poznan University of Medical Sciences, Poznan, Poland; ^4^Department of Pediatric Oncology, Hematology and Transplantology, Poznan University of Medical Sciences, Poznan, Poland

**Keywords:** antifungal treatment, child, fungal infection, leukemia, mucormycosis, surgery

## Abstract

Mucormycosis is a rare but a devastating and lifethreatening fungal infection caused by fungi of the order Mucorales usually in immunocompromised patients. Depending on the organs and tissues involved, there are sinus, pulmonary, gastrointestinal, orbital, cerebral, cutaneous and disseminated mucormycosis. Only sporadic cases of hepatic mucormycosis have been described. Hence, we present a complicated treatment management in a 16-month-old child with leukemia and generalized mucormycosis localized in the liver and in the gastrointestinal tract. The collaboration of a multidisciplinary team and appropriate therapy gave a chance not only to save the patient's life, but to carry out anticancer treatment, which resulted in leukemia remission. A 6-month course of isavuconazole and amphotericin B liposomal as well as surgical treatment led to the cure of the fungal infection.

## Introduction

Mucormycosis is a rare disease caused by fungi of the order Mucorales. After aspergillosis and candidiasis, it is the third most common invasive fungal disease, occurring mainly in oncology and transplantation units' patients (1, 2). Infection in humans can be caused by different types and species, the most frequently found are Rhizopus arrhisus, Lichtheimia spp., Rhizopus microsporus, Mucor spp. Depending on the organs and tissues involved, there are sinus, pulmonary, gastrointestinal, orbital, cerebral, cutaneous and disseminated mucormycosis (3–5). Only sporadic cases of hepatic mucormycosis have been described (6, 7).

These fungi are very common in our environment. Fungus spreads by spores of molds of the order Mucorales, most often through contaminated food, inhalation, or contamination of open wounds (6). These fungi are common in soils, but usually do not affect people (8). Risk factors include diabetes, leukopenia, cancer, organ transplant, iron overload, problems with kidney, long-term steroids or use of immunosuppressants, and to a lesser extent in HIV/AIDS (1, 5).

Mucormycosis is a devastating and lifethreatening fungal infection. Mucormycosis tends to progress rapidly and leads to death in about half of sinus infection cases and in almost all cases of generalized infection (3, 9). Diagnosis requires biopsy and culture (10). Surgical treatment in addition to antifungal drugs is an important component of the therapeutic management of mucormycosis. (7).

The purpose of our study is to present a complicated treatment management in a 16-month-old child with leukemia and generalized mucormycosis localized in the liver and in the gastrointestinal tract. The collaboration of a multidisciplinary team and appropriate therapy gave a chance not only to save the patient's life, but to carry out anticancer treatment, which resulted in leukemia remission. A 6-month course of isavuconazole and amphotericin B liposomal as well as surgical treatment led to the cure of the fungal infection.

## Case Report

A 16 months old girl was admitted to the hospital with suspected leukemia. Two months before admission, the child had undergone SARS-CoV-2 infection. Child from 2nd pregnancy, 2nd vaginal delivery, without chronic diseases. Patient was up to date on routine childhood immunizations as per Polish vaccination schedule. Older sibling, parents and grandparents do not have chronic illnesses, there are no oncological diseases in the family.

Acute lymphoblastic leukemia, precursor B cell type without central nervous system involvement was diagnosed and the child started chemotherapy acc. to AIEOP-BFM ALL 2017 protocol. No translocations in blast cells were identified and the karyotype of the leukemic cells was normal.

**Figure 1:**
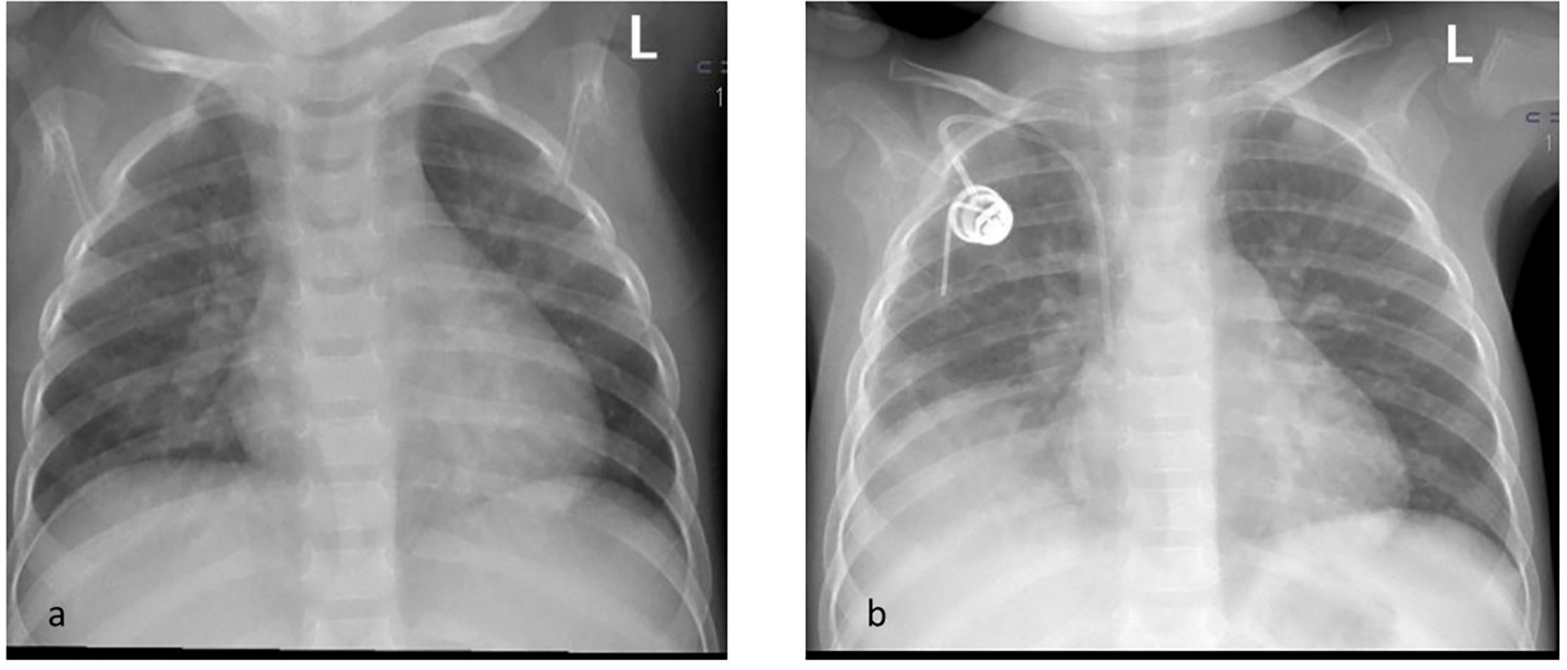
Chest x ray on the day of admission **(a)** showing peribronchial cuffing and after 3 monthes **(b)** with consolidation of right mid and lower zones.

Physical examination on admission showed no abnormalities. Palpation and ultrasonography did not reveal hepatosplenomegaly. Chest X-ray showed peribronchial cuffing (Figure 1).

**Figure 2:**
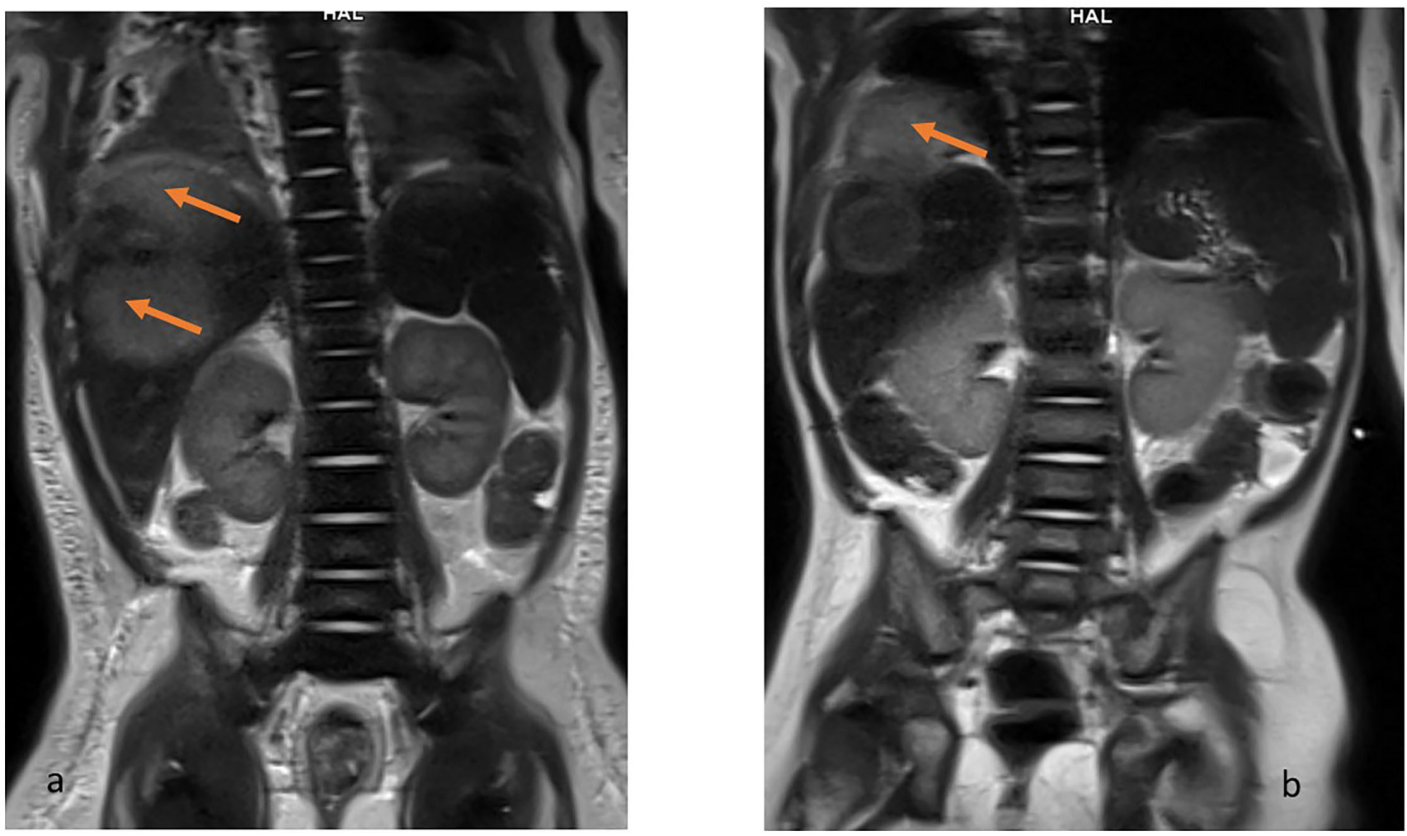
MRI T2-weighted HASTE coronal view. Hiperintense lesions in the right lobe of liver **(a)** and the examination after 2 months when the lesion exceeds the limits of the diaphragm and penetrates the chest **(b)**.

**Figure 3:**
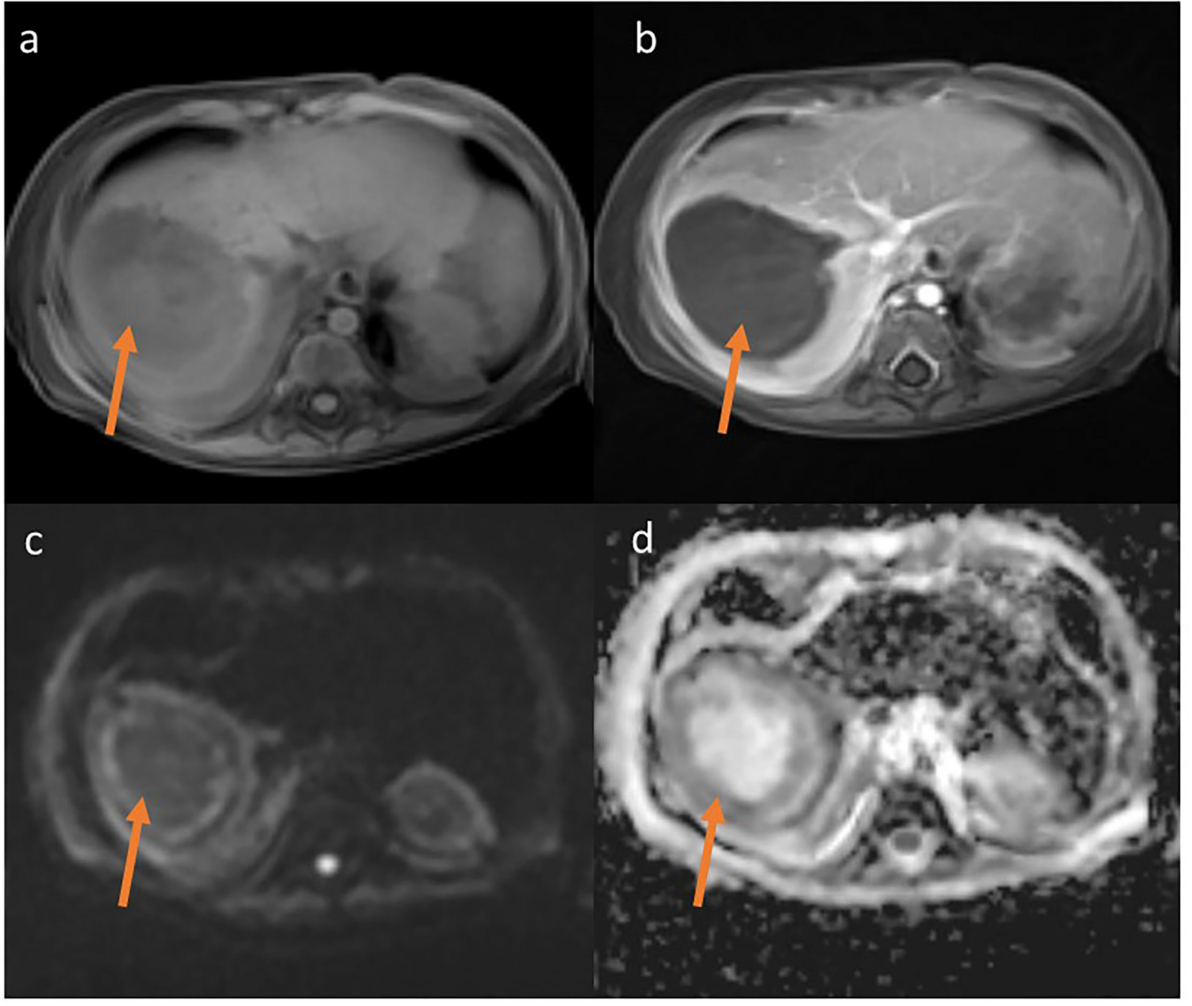
MRT T1-weighted Starvibe FS, hipointense lesions in right lobe of liver **(a)** T1-weighted Starvibe FS post contrast-nonenhancing lesion **(b)**, DWI b800 **(c)** ADC **(d)**-peripheral diffusion restriction (examination performed before surgery).

Treatment in the first week was complicated by rotavirus infection. During the second week of hospitalization, a perianal abscess appeared, and after a further 5 days, the child began to have a fever, the liver enlarged by 3 cm. Ultrasonography and magnetic resonance imaging (MRI) (Figures 2, 3) revealed a well-demarcated, hypoechoic area in segment VII of the liver.

The lesion measured 32 x 27 mm, with no detectable vascular flow on CD or SMI. A similar lesion was located at the border of segments VI and VII (16 x 17 mm, border 4 mm) and two other adjacent lesions in segment V (or one hourglass-shaped) measuring 17 x 17 mm and 12 x 9 mm. Additionally, an irregular, extensive parenchymal area with increased echogenicity was observed at the border of segments VI and VII. The ultrasound image of the lesions indicated multiple liver abscesses. In the subcutaneous tissue of the rectal region on both sides, hyperechoic areas surrounded by a hypoechoic “halo” with strongly increased vascular flow registered in SMI examination were observed (features of inflammation). The lesion on the right side measured 8.5 x 2.5 x 19 mm, on the left 7.5 x 2.5 x 18 mm.

**Figure 4:**
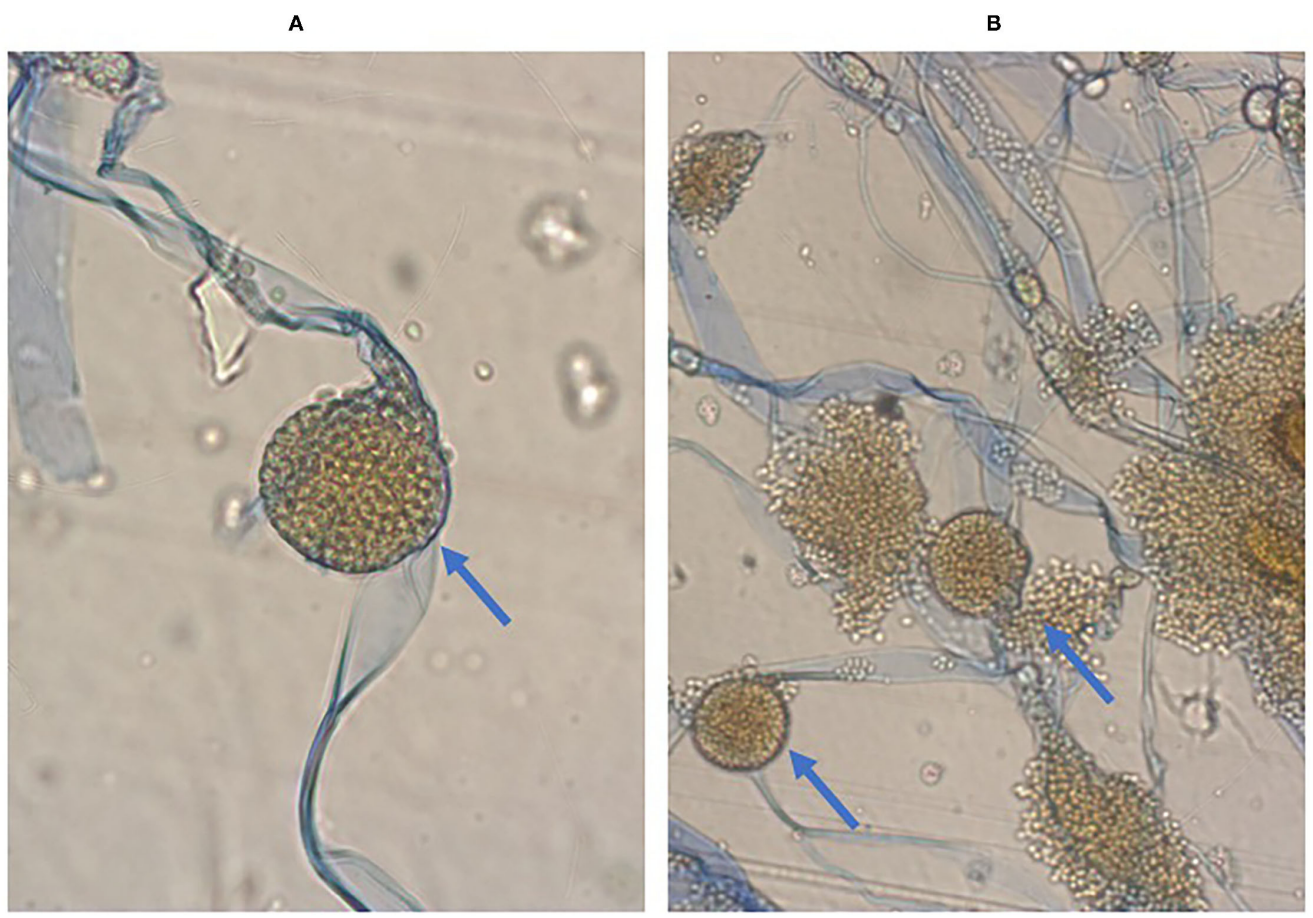
Microscopic image of Rhizopus microsporus. Stipules broad, sporangiophores non-branching, sporangia located apically, rhizoids present, Preparation from culture on SDA+ chloramphenicol medium. Magnification 400x, Lactophenol Cotton Blue staining **(A)**. Seven-day old Rhizopus microsporus colony fluffy, light gray with age, darkening, fast growing. Preparation from culture on SDA+ chloramphenicol medium. Magnification 400x, Lactophenol Cotton Blue staining **(B)**.

A biopsy of the liver lesion and perianal abscess was performed. Histological examination with Grocott's staining showed fungal infection with Mucor spp. Rhizopus microsporus was identified in culture (Figure 4).

According to the recommendations, the dosage of liposomal amphotericin ranges from 5–10 mg/kg per day iv and isavuconazole 10 mg/kg per day iv (every 8 h on day 1-2) (11–13). In our patient, the dosage of amphotericin was 5 mg/kg per day iv for the first 3 days, then the dose was increased to 10mg/kg per day iv. Isavuconazole was administered at a dose of 10mg/kg/day.

**Figure 5:**
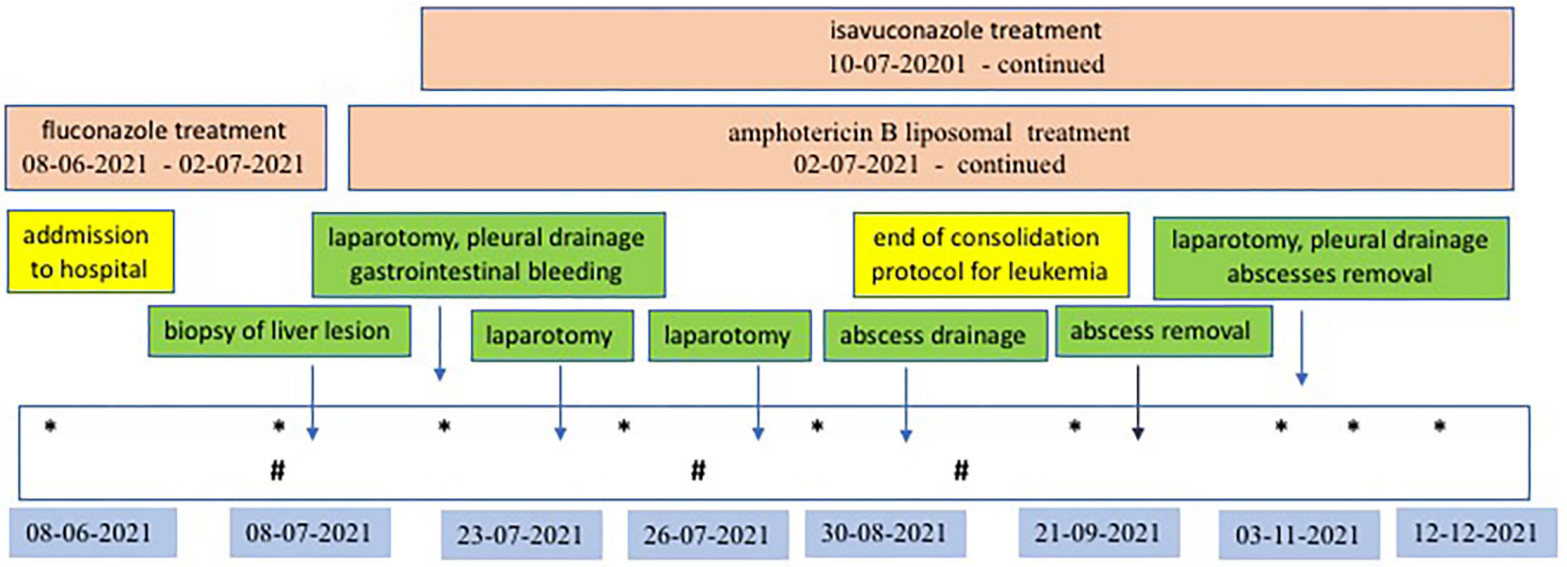
Antifungal and surgical treatment scheme. *Ultrasonography examination of abdomen and lungs; ^#^MR examination of abdomen.

Despite the amphotericin B liposomal treatment further progression of the fungal infection was observed. The child developed massive bleeding from the upper small intestine due to infiltration of the intestinal wall by the fungus. The girl underwent repeated gastroscopy and colonoscopy, pleural drainage was necessary. Two exploratory laparotomies had to be performed, during which a significant amount of blood was found inside the small intestine, but no specific bleeding site was identified. The child's condition slowly improved, and the use of a combination of antifungal treatment with two drugs isavuconazole and amphotericin B liposomal resulted in absorption of the perianal abscess and reduction of the liver abscesses. It also allowed the leukemia to be treated by day 100 of the protocol and achieve cytological and molecular remission on 33rd day of the protocol. Five months after admission, another laparotomy was performed, during which the lesions–liver abscesses, were finally radically removed. They were hard, consolidated and markedly limited. One of the lesions infiltrated the diaphragm, so a fragment of the diaphragm must be removed. Above the diaphragm, an inflammatory changed, fibrotic lung was visualized, which was also resected. The histopathological examination of the removed lesions as well as culture confirmed Rhizopus microspores. The detailed treatment plan, examinations and procedures performed are shown in Figure 5.

Currently, the child remains in full remission of leukemia and continues to receive antineoplastic and antifungal therapy only with oral isavuconazole.

## Discussion

Mucormycosis was first diagnosed in patient with diabetes by Paultauf in 1885 (14). Usually as a life-threatening invasive infection, mucormycosis is now seen in immunocompromised patients (7). The 16-month-old girl we treated developed generalized mucormycosis at the beginning of therapy for acute lymphoblastic leukemia, during intensive treatment with cytostatics and steroids.

The diagnosis of mucormycosis remains challenging and relies on recognition of risk factors, evaluation of clinical symptoms, early use of imaging studies including CT and MRI, and prompt initiation of diagnosis. The clinical manifestations of mucormycosis are nonspecific, often limited to fever in the initial phase. Fungal infections are predisposed primarily by immunocompromised states, neutropenia, especially prolonged >9 days, and lymphopenia (11). They may result from oncological disease and previous treatment–chemotherapy, steroid therapy or radiotherapy. The risk of fungal infection is also increased by the use of intravenous catheters, abdominal or thoracic surgery, gastrointestinal mucositis and skin lesions, history of respiratory infections. A history of voriconazole prophylaxis and the appearance of an active fungal infection alone suggests the presence of mucormycosis. Another sign suggestive of mucormycosis is the reverse halo sign (RHS) on CT imaging. However, definitive diagnosis requires histopathology and culture (11, 12).

Histopathology, direct microscopy, and culture remain the primary tools, although serologic and molecular methods are increasingly applicable. In the direct microscope, Mucorales produce unpigmented, broad (5–20 μm), thin-walled, ribbon-like hyphae without septa, with right-angled branching, in contrast to the Aspergillus, which are typically 3–5 μm wide with septa and acute-angled branching (11, 13). Routine hematoxylin and eosin (H&E) staining and the much more effective Grocott methenamine-silver (GMS) and periodic acid (PAS) staining are used for diagnosis. In our material, the preparation was stained with both H&E and PAS. Immunohistochemical studies using monoclonal antibodies are also useful in differentiating aspergillosis from mucormycosis. Mucorales grow on any carbohydrate medium such as Sabouraud agar and potato dextrose agar incubated at 25–30°C, and identification is based on colony morphology and microscopy. However, the main problem with culture is its low sensitivity. The culture can be falsely negative in up to 50% of cases of mucormycosis. In conclusion, direct microscopy of fresh material next to histopathology and culture is recommended as a diagnostic standard by the expert panel of the European Confederation of Medical Mycology (11, 13).

In neutropenia, Mucorales spores grow uncontrolled. Angioinvasion, which is the distinctive feature of Mucorales (3), leads to vascular thrombosis and necrosis of the involved tissues, which may result in difficulties in obtaining adequate biopsy material, on the one hand, and insufficient penetration of antifungal drugs into the involved tissues and progression of the disease. In patients with severe immunosuppression, Mucorales predominantly affects the lungs, nasal sinuses, and orbital and brain tissue. Less commonly, abdominal organs and skin are also involved. Based on the location, there are 6 main forms of mucormycosis: nasal-brain, pulmonary, cutaneous, gastrointestinal, disseminated, and miscellaneous (with endocarditis, osteomyelitis, and kidney infection) (15, 16). Studies show that the most common locations of mucormycosis are sinus (39%), lung (24%), skin (19%), brain (9%), gastrointestinal (7%) and kidney (2%), and disseminated infection (3%) (3). Our patient presented symptoms of disseminated mucormycosis localized in the liver and in the gastrointestinal tract.

The clinical picture of pulmonary mucormycosis can vary depending on the immune status of the patient. Symptoms include fever, cough, chest pain and dyspnea (17). On imaging studies, pulmonary mucormycosis does not produce specific findings. It may manifest as a single nodule, lobular consolidation as in pneumonia, cavitary lesion or disseminated appearance (18). The clinical and radiological manifestations of pulmonary mucormycosis were not confirmed in our patient.

Gastrointestinal mucormycosis can occur at any site of the gastrointestinal tract. The most common location is the stomach (58%), followed by the large intestine (32%), ileum, and esophagus (19, 20). Symptoms of this form of mucormycosis include pain, spreading sensation, fever, hematemesis and hematochezia due to bleeding. Gastrointestinal lesions manifest as ulceration and can lead to intestinal perforation and bleeding, peritonitis, sepsis, and hemorrhagic shock. Our patient developed massive upper small bowel bleeding. At gastroscopy, colonoscopy, and double laparotomy, the small bowel mucosa was inflamed, necrotic, and bleeding. Repeated transfusions of blood products and long-term pharmacotherapy with etamsylate and tranexamic acid were necessary. However, combinations of multiple surgical treatments and two-drug therapy were crucial for the cure of this fungal infection.

Mucormycosis confined to the liver is extremely rare. Usually, multiple liver lesions present as disseminated mucormycosis (7). In our patient, liver mucormycosis presented as multiple abscesses. There is little evidence in the available bibliography to support the superiority of any of the pharmacologic options for the treatment of mucormycosis. There are several potentially effective drugs for the treatment of mucormycosis. These include posaconazole, isavuconazole, amphotericin B or its liposomal form, and combination treatment with these agents (7). Hepatic mucormycosis can be treated with antifungal drugs alone or in combination with surgery (21). Long-term therapy with isavuconazole and amphotericin B liposomal in our patient resulted in reduction and consolidation of the liver lesions. The single liver abscess was drained through the abdominal wall, and the remaining lesions, had to be removed during laparotomy.

When Mucormycosis is suspected, immediate initiation of effective antifungal therapy in conjunction with appropriate surgical management and, if possible, control of underlying predisposing factors is strongly recommended. Liposomal amphotericin B and amphotericin B lipid complex are first-line treatments in all age groups. Rescue or continuation treatment options include isavuconazole and posaconazole in children ≥13 years of age (11, 12). The available literature on the treatment of mucormycosis is limited, but there are examples for the successful treatment with isavuconazole alone (22) or in combination therapy of liposomal amphotericin with posoconazole or isavuconazole (11, 12, 23). In our patient, the high dosage of amphotericin was used (10mg/kg per day iv).

Data are also lacking on the length of isavuconazole therapy, although treatment of coccidioidal meningitis has ranged from 274 to as long as 810 days without significant toxicity (24). The toxicity of isavuconazole in hematologic patients was evaluated in several studies, indicating good tolerability of therapy lasting up to 24 months (11, 25). No toxicity was observed in our patient during 6-month combination treatment with two antifungal drugs. Also, no toxicities are currently observed during oral treatment with isavuconazole alone, despite further treatment with chemotherapy.

Disseminated mucormycosis is associated with a very high mortality rate of nearly 100%, but successful treatment has also been reported (26, 27). The cooperation of a multidisciplinary team and the use of all possible forms of therapy gave a chance not only to save the patient's life, but to carry out anticancer treatment, which resulted in leukemia remission. A 6-month course of isavuconazole and amphotericin B liposomal as well as surgical treatment led to the cure of the fungal infection.

## Conclusions

Mucormycosis is a life-threatening infection in immunocompromised patients. Multidisciplinary teamwork and complex therapy offer the chance to save the patient's life. There is no clear-cut single most effective form of treatment in mucormycosis–to save lives, a combination of two anti fungal medication and repeated surgery may be necessary, but the safest form of treatment should be guided by the clinical condition.

## Data Availability Statement

The raw data supporting the conclusions of this article will be made available by the authors, without undue reservation.

## Author Contributions

PS-S, KB, HK-R, PM, and DJ-L: conceptualization, methodology, validation, formal analysis, resources, data curation, and administration. PS-S, KB, and HK-R: software. PS-S: investigation and writing—original draft preparation. DJ-L: writing—review and editing. KB, HK-R, PM, and DJ-L: visualization. PM and DJ-L: supervision. All authors have read and agreed to the published version of the manuscript.

## Conflict of Interest

The authors declare that the research was conducted in the absence of any commercial or financial relationships that could be construed as a potential conflict of interest.

## Publisher's Note

All claims expressed in this article are solely those of the authors and do not necessarily represent those of their affiliated organizations, or those of the publisher, the editors and the reviewers. Any product that may be evaluated in this article, or claim that may be made by its manufacturer, is not guaranteed or endorsed by the publisher.
